# A Successful Pregnancy Outcome in a Human Immunodeficiency Virus Serodiscordant Couple in a Single Cycle of In Vitro Fertilization/Intracytoplasmic Sperm Injection: A Case Report

**DOI:** 10.7759/cureus.29981

**Published:** 2022-10-06

**Authors:** Achyut Wadkar, Akash More

**Affiliations:** 1 Anatomy, Jawaharlal Nehru Medical College, Datta Meghe Institute of Medical Science, Wardha, IND

**Keywords:** infertility, pcos, art, haart, semen wash

## Abstract

Human immunodeficiency virus (HIV) is a virus that affects the immune system and attacks immune cells called CD4 while also raising the risk and severity of other infections and diseases. Despite the fact that HIV can infect persons of any age, the majority of infected people are those of reproductive age between 15 and 44 years. Many women and men with HIV desire children. Plenty of HIV-positive adults worldwide wish to have a child. As a result, reproductive desires have emerged as clinically significant in patients with HIV/acquired immunodeficiency syndrome (AIDS). We present a case of a 35-year-old male who is HIV-1 seropositive and his 32-year-old healthy wife who is seronegative who visited an in vitro fertilization (IVF) clinic. The couple was married for four years, facing primary infertility, and had a history of failure of three successive intrauterine insemination (IUI) cycles. The couple abstained from physical contact when the husband was discovered HIV positive. The couple wanted to have their own biological, completely healthy child. The male was undertaking highly active antiretroviral therapy (HAART). On the performance of the semen analysis test, the male was discovered to have oligospermia. The blood test of the female revealed that her anti-Mullerian hormone (AMH) level was raised, and ultrasound indicated polycystic ovarian syndrome (PCOS). The couple was suggested IVF/intracytoplasmic sperm injection (ICSI) treatment due to failure of IUI cycles in the past. Medications were given to the wife for her PCOS. Self-oocytes were retrieved from the wife, and the husband’s semen sample was washed with the semen washing method (density gradient (DG) and swim up). By confirming in the sample that HIV is not present, ICSI was done. Three blastocyst stage embryos were transferred on day 6 of progesterone. After 14 days, the test report of the β-human chorionic gonadotropin (hCG) hormone came positive. Around 39 weeks later, she successfully delivered a baby boy who was later tested for HIV, and the report was negative.

## Introduction

Human immunodeficiency virus/acquired immunodeficiency syndrome (HIV/AIDS) is a worldwide health issue that has resulted in the infection of nearly 70 million individuals, the death of 35 million people, and the ongoing affliction of 36.7 million people. Human immunodeficiency virus (HIV) is a virus that lowers the immune system’s defenses against common infections and diseases [1]. The global AIDS epidemic is brought on by HIV type 1 (HIV-1) and HIV type 2 (HIV-2) lentiviruses, which differ genetically. The considerable genetic heterogeneity of the human immunodeficiency virus (HIV) is one of the challenges in treating it. There are two main forms of HIV: HIV type 1 and HIV type 2. The most prevalent and dangerous form of the virus is HIV-1. More than two million of these infections occur every year. HIV-1 was categorized into a major group (Group M) and two or more minor groups (Group N, Group O, and Group P) [2]. Around the 1920s, in the modern-day Democratic Republic of the Congo, near Kinshasa, HIV-1 is believed to have first appeared. From there, it proliferates via a transit network to other regions in sub-Saharan Africa, West Africa, Europe, and the rest of the world [3]. Most frequently, the virulence of HIV-1 is determined by two methods: viral loads (the number of viral particles in blood plasma) and CD4 counts (the quantity of CD4+ T-cells in peripheral blood, which monitors immune system damage caused by the virus). By keeping viral loads below detection levels, highly active antiretroviral therapy (HAART) has considerably decreased the mortality rate and prolonged the life duration of individuals who are infected by HIV. This has prevented the onset of AIDS. HIV patients’ psychosocial characteristics may impact their reproductive goals and outcomes [4].

Biological changes in reproductive physiology may cause subfertility in HIV-positive people. HIV-positive couples also wish to have healthy children. Couples who have had intercourse for at least a year without birth control and are still unable to conceive are said to be experiencing infertility [5]. Using assisted reproductive techniques (ART), specific in vitro fertilization (IVF) clinics started treating couples where one person has HIV for a few years to limit the risk of transmission to the unaffected partner or address any fertility issues that may already present. In the case of a male HIV-infected partner, “sperm wash” is ideal for washing the semen sample and removing the virus of HIV. It is widely used and considered a safe method. Washing of semen is beneficial in intrauterine insemination (IUI), IVF, and intracytoplasmic sperm injection (ICSI) procedures. A total of 85 HIV-discordant couples underwent reproductive screening, out of which 29 uninfected female partners did not contract HIV in the first study in 1989 that offered washing of semen to HIV-serodifferent partners using intrauterine insemination (IUI) [6].

## Case presentation

This case report shows a 32-year-old female facing primary infertility for the last four years. She visited an IVF clinic with her husband (35 years old) to treat her infertility in January 2021, and she was enrolled for three cycles of IVF. The female was affected by polycystic ovarian syndrome (PCOS). She was suffering from secondary amenorrhea (anovulatory infertility). The male was HIV-1 seropositive and also had a low sperm count (oligospermia). The male was diagnosed with HIV infection after marriage. He was undertaking highly active antiretroviral therapy (HAART), and since then, the couple has avoided having intercourse to have a healthy baby. The couple visited the IVF clinic in Nanded District, Maharashtra, India. The couple had no habit of eating tobacco, consuming liquor, or smoking on daily basis.

Medical history

**Figure 1:**
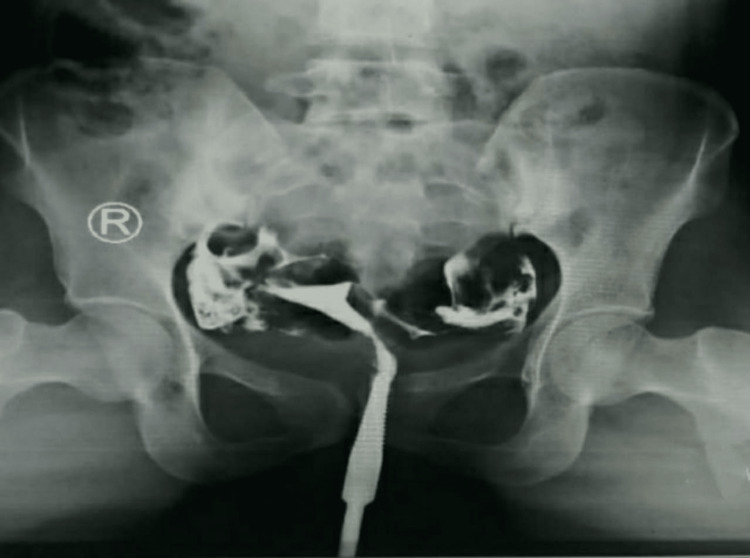
Bilateral Spillage of Contrast in Hysterosalpingography (HSG)

The female underwent routine hysteroscopy. Her uterine cavity was normal, both ostia were visualized, and the cervix and endocervix were also normal. The female had undergone hysterosalpingography (HSG) in 2020 and showed no presence of any blockage. The spillage of bilateral peritoneal contrast was seen (Figure 1). Proper oral and written consent from the female was taken before the procedure. The couple underwent an IUI procedure thrice, in which the semen sample from the husband was taken and centrifuged with media. The debris material and dead sperms were discarded. Highly motile sperms were extracted and transferred to the uterus of the female. However, all the attempts of IUI failed. The male patient was HIV-1 seropositive, and the female had PCOS. They do not have any family history of major diseases or surgeries and did not possess any history of mental disorders as well.

 Clinical findings

**Figure 2:**
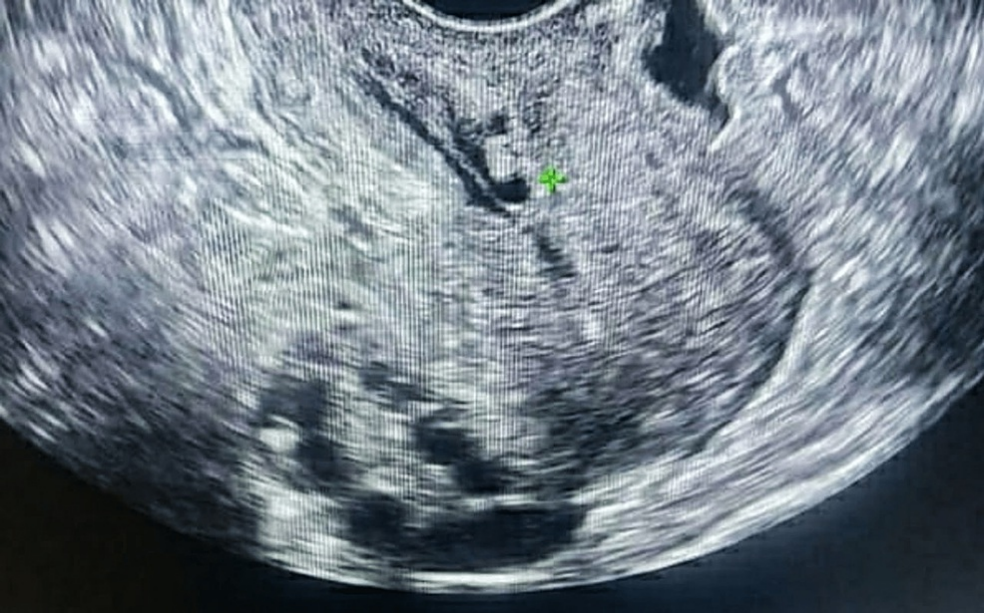
Formation of Multiple Cysts on the Ovary Showing Polycystic Ovarian Syndrome (PCOS)

The female had an average body temperature. Her blood pressure was normal (118/78), pulse rate was 73 beats/minute, weight was 60 kg, and height was 5 feet and 2 inches. The male had average body temperature. His pulse rate was 88 beats/minute, blood pressure was 130/80 mmHg, weight was 70 kg, and height was 5 feet and 5 inches. All of these are one-time findings of the female and male patients during treatment. Ultrasonography of the female’s abdomen and pelvis showed hypertrophy of bilateral ovaries and the presence of follicles on both ovaries (Figure 2). Due to PCOS, her menstruations were absent, and her last menstruation date was on October 10, 2020 (before three months). The male was discovered as oligospermic on a semen analysis test (the sperm count of the male was 12 million/mL). The concentration of sperms was below 15 million as per the latest WHO guidelines, which is considered oligospermia.

Timeline

The couple visited our IVF clinic in the month of January 2021. The couple enrolled in the same month of January 2021 and started treatment for primary infertility. Due to the failure of three successive IUI cycles, they were suggested to go for direct IVF/ICSI treatment. The female was stimulated (controlled ovarian stimulation) for around 12 days. In the month of March 2021, ovum pickup was done, and 05 MI and 06 MII oocytes were retrieved. ICSI was done with the husband’s washed and HIV (absent)-tested sperm sample. Morphologically good-quality sperms were injected into the oocytes. Three embryos on day 5 were formed and frozen. On the day of embryo transfer in May 2021, the thawing of these embryos was done using the Kitazato thawing kit (VT 601, Japan). These embryos were kept in a Minc Trigas benchtop incubator (Cook) for expansion. These embryos are also called blastocysts. After around two hours of thawing, three blastocysts of 4AA quality were transferred. This was her first embryo transfer. On day 14, the β-human chorionic gonadotropin (hCG) test was done, which came positive. After 39 weeks of pregnancy, she successfully delivered a healthy baby boy.

Diagnostic assessment

Laboratory Findings of the Female

The hemoglobin (Hb) value was 11.08 mg/dL. The follicle-stimulating hormone (FSH) level was 5.65 mIU/mL. The luteinizing hormone (LH) level was 9.40 mIU/mL. The anti-Mullerian hormone (AMH) test level was 16.08 ng/mL. The thyroid-stimulating hormone (TSH) level was 2.37 µIU/mL. The prolactin level was 24.88 ng/mL. Reverse transcription-polymerase chain reaction (RT-PCR) was negative.

Laboratory Findings of the Male

The hemoglobin (Hb) value was 12.03 mg/dL. The cluster of differentiation 4 (CD4)+ T-cells were more than 300/mm^3^ (tested thrice at least in six months). The viral load in stable blood (titer of ribonucleic acid (RNA)) is less than 60 copies/mL more than thrice consecutively during a period of four months. The serum CD4 T-cell count was 910/mm^3^. The semen analysis report consists of a sperm count of 12 million/mL, total motility of 65% motile, and 10% normal sperm morphology. In a seminal fluid, the viral load of RNA was less than 1,000 copies/mL.

Therapeutic intervention

Daily exercise, a healthy diet containing low sugar, and maintenance of low weight (so that the body mass index (BMI) range will be between 18.5 and 24.9 kg/m^2^) were suggested to the female patient for her PCOS. Once a day, metformin 500 mg was given to the patient, and the dose was raised gradually. Clomiphene citrate 50 mg was given for around one week to have regular periods.

Complete procedure regarding washing of semen sample was done in microbiological safety cabinet in our clinic using the universal precautions. A fresh semen sample was collected from the husband. The density gradient (DG) process was carried out to wash the sperms inside a conical tube containing 1 mL 80% and 1 mL 40% DG media overlaid. The 2 mL semen sample was again overlaid on it. The tube was centrifuged for 15 minutes at 1,500 rpm. The supernatant was discarded, and in the pellet, 3 mL of wash medium was mixed and centrifuged for five minutes at a thousand rpm. The supernatant was discarded, and 0.5-1 mL wash medium was added to the pellet. This is called the swim-up method. The tube was tilted for 45° angulation for at least two hours. After this, the highly motile sperms will travel toward the surface and be taken out. This supernatant sample was sent for an HIV RNA test. HIV-1 RNA was not detected in the sample. Using this sample, ICSI was done, and the remaining sample was frozen for further use.

Follow-up and outcomes

Follow-up is necessary for the patients. During ovum pickup, 05 MI and 06 MII oocytes were retrieved. ICSI was done with the self-sample from the husband. The endometrium is measured at each checkup to ensure proper and successful implantation of the embryo in the uterus. This measurement also allows for the observation of the steady growth in endometrial thickness. Before beginning medication for embryo transfer, the endometrium thickness must be less than 5 mm. On the day of embryo transfer, three day 5 embryos were transferred. Later on the 14th day, a β-hCG test was done, which was positive.

## Discussion

In 2021, 38.4 million (33.9 million-43.8 million) individuals were suffering from HIV worldwide, 1.5 million (1.1 million-2 million) persons contracted HIV for the first time, and 650,000 (510 000-860,000) persons passed away from AIDS-related diseases. People of all ages can contract human immunodeficiency virus (HIV). People of reproductive age with HIV, many of whom express a desire for biological parenting, are affected by this dangerous but treatable chronic disease [7]. The most prevalent form of HIV is HIV-1. The immune system in the body is attacked. Viruses obliterate CD4 cells. The body uses these cells to combat illnesses. Acquired immune deficiency syndrome (AIDS) and severe immune system damage from HIV-1 are both possible consequences [8]. Since 2004, there have been 940,000 fewer fatalities from opportunistic diseases of the acquired immunodeficiency syndrome than in 2017. This has been made possible, in part, by using antiretroviral therapy (ART) and highly active antiretroviral therapy (HAART). Many medications that act on various viral targets have been used, and they are known as highly active antiretroviral therapy. The overall load or pressure of HIV patients is decreased by HAART, which also maintains the proper function of the immune system, and guards against opportunistic infections, which frequently result in fatalities [9]. As long as the HIV-positive partner maintains an undetectable viral load, HAART also prevents HIV transmission between serodiscordant partners who engage in both same-sex and opposite-sex relationships. However, in routine clinical practice, HAART success, which is defined as the suppression of detectable plasma virus and recovery/increase in CD4+ cell counts after 6-12 months, does not surpass 40%-60% of treated patients, typically regardless of the regimen selected. Newly diagnosed HIV/AIDS patients are now managing a chronic condition because of the effectiveness of the treatment. Approximately one-third of people of reproductive age with HIV want to have children. Reproductive aspirations have thus become clinically significant in HIV/AIDS patients [10].

Many HIV serodifferent couples are so desperate to begin a family to have their child that they are willing to forego condom-protected sex despite the hazards. Washing of semen is a safe procedure used in the reproductive technique that male-infected HIV couples can use to conceive. The sperm fractions that are HIV negative are used in assisted reproduction after spermatozoa, which are not HIV vectors, are removed during semen washing from the seminal fluid present in the surrounding [11]. The use of IUI with stimulation of ovaries or natural cycles, IVF, or intracytoplasmic sperm injection were all examples of assisted reproductive technologies, in which the semen washing method is used. Some claim that IVF and IVF/ICSI produce superior reproductive results than IUI. However, in the HIV-discordant group under consideration, the overall pregnancy success rates were comparable amongst the techniques. Furthermore, some claim that IVF or IVF/ICSI only uses one spermatozoon to reduce the risk of HIV transmission compared to IUI [12]. Testing in the laboratory showed that cells from HIV-1 infection from the HIV serum-positive men’s semen might be successfully extracted by gradient centrifugation, followed by a swim-up method. Semen washing and assisted reproduction have historically been utilized to address the needs of infertile or subfertile couples. The effect of infertility was seen in one in four couples in underdeveloped nations, according to a 2012 WHO study. According to this survey, there were 48.5 million couples who are heterosexually stable and have been trying to get pregnant for five years or more who were infertile worldwide, of which 10 million were in sub-Saharan Africa, the area most afflicted by HIV [13].

Our patient was an HIV-1 seropositive who was undertaking HAART; some research has suggested that the drugs used in this therapy may affect reproductive health, including the concentration, motility, and morphology of sperms [14]. Our patient also has a low sperm count (oligospermia), and the morphology was affected. It was suggested that for HIV patients who are taking antiretroviral therapy, the chances of transmission of HIV were decreased from HIV-seropositive husband to his wife through unprotected intercourse. However, the transmission rate was not ultimately disappeared, although it is less. In such cases, assisted reproductive technique (ART) plays a crucial role, which is a blessing for such patients. ART helps HIV couple have their child [15]. Semen washing is a way to have a healthy child for HIV-positive males. In this process, semen is washed through the density gradient and swim-up method. This helps in the extraction of highly motile sperms and the removal of dead sperms, non-sperm cells, and debris from the seminal plasma, which contains the virus. The method of washing semen is now widely used in ART laboratories. Even in ART procedures, ICSI is considered the safest procedure because it uses a single sperm to insert into an oocyte compared to other procedures such as IUI and IVF, and that is the reason we used the ICSI procedure due to the failure of previous IUI cycles [16].

## Conclusions

There are various studies that suggest that semen washing prevents the transmission of HIV in couples who are trying to have a child where the male partner is HIV-infected. Assisted reproductive technique plays a significant role for HIV serodiscordant couples. We believe that IVF/ICSI is the ideal and safe choice for HIV serodiscordant couples to fulfill their wish of having a baby.
